# Incidental Detection of a Benign Thymoma on Tc-99m MIBI Myocardial Perfusion Study

**DOI:** 10.4274/MIRT.019513

**Published:** 2011-08-01

**Authors:** Funda Aydın, Evrim Sürer Budak, Levent Dertsiz, Aytül Belgi, Gökhan Arslan, Fırat Güngör

**Affiliations:** 1 Akdeniz University Medical School, Department of Nuclear Medicine, Antalya, Turkey; 2 Akdeniz University Medical School, Department of Thoracic Surgery, Antalya, Turkey; 3 Akdeniz University Medical School, Department of Cardiology, Antalya, Turkey; 4 Akdeniz University Medical School, Department of Radiology, Antalya, Turkey

**Keywords:** Thymoma, technetium Tc 99m sestamibi, myocardial perfusion imaging

## Abstract

Technetium-99m methoxy-isobutylisonitrile (Tc-99m MIBI) is a routinely used radiopharmaceutical for myocardial perfusion scintigraphy (MPS). It is also a tumor seeking agent. Here, we present a case of 51 year old male who underwent Tc-99m MIBI myocardial perfusion study due to permanent chest pain after coronary angiography. Abnormal MIBI uptake in the thorax was detected in the raw images. This single finding led to further investigation and thoracotomy proved that the lesion was benign thymoma. Thymomas are often asymptomatic or have a non-specific presentation. They are often detected coincidentally on images performed for any other reasons. We wanted to emphasize that during of MPS, the raw data should always be reviewed as occasionally valuable additional information on noncardiac pathology could be recognized by extracardiac uptake, as in this case.

**Conflict of interest:**None declared.

## INTRODUCTION

Technetium-99m methoxy-isobutylisonitrile (Tc-99m MIBI) is a routinely used radiopharmaceutical for myocardial perfusion scintigraphy (MPS). It is also a tumor seeking agent(1,2,3). Here, we present a case of 51 year old male who underwent Tc-99m MIBI myocardial perfusion study due to permanent chest pain after coronary angiography and abnormal MIBI uptake in the thorax was detected in the raw images.

## CASE REPORT

A 51 year old man with permanent chest pain after coronary angiography was referred for myocardial perfusion scintigraphy (MPS). He had undergone coronary angiography three months ago and coronary artery stenting had been performed. A standard 2-day protocol of Tc-99m MIBI MPS was performed. Stress and rest MPS showed an abnormal focus of tracer uptake at the upper side of the heart within the mediastinum (Figure 1a and 1b). 

**Figure 2:**
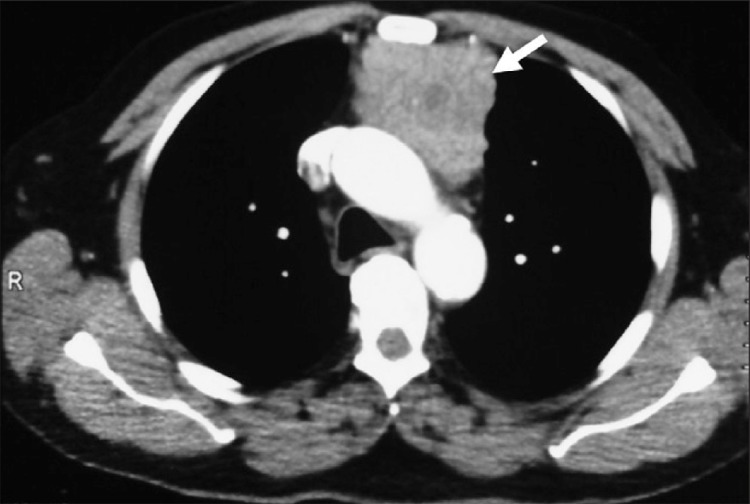
Contrast-enhanced thorax CT shows soft tissue mass in theanterior mediastinum (arrow)

**Figure 3:**
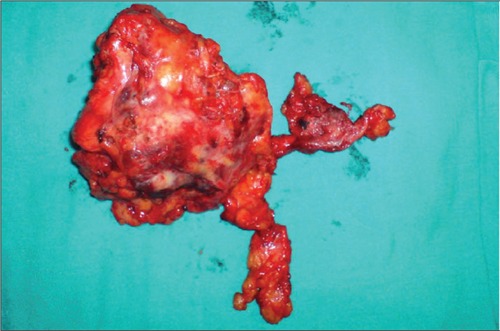
Surgical specimen. The mass is 9 cm in its greatestdiameter with no pericapsular fat tissue invasion

For further investigation, thoracal computerized tomography (CT) was also perfomed. Thorax CT demonstrated a 5.5x1.5 cm soft tissue mass with parenchymal calcifications in the anterior mediastinum with no evidence of local invasion (Figure 2). There was no significant hilar or mediastinal lymphadenopathy. The patient was referred to thoracic surgery and thoracotomy was performed. The mass was 9 cm in its greatest diameter with no pericapsular fat tissue invasion (Figure 3). Histopathologic examination of the surgical specimen revealed thymoma, type AB.

## LITERATURE REVIEW AND DISCUSSION

Thymic neoplasia is the most common cause of an anterior mediastinal mass (1). Thymoma has distinct clinicopathological features and the World Health Organization divides them into six categories. The first two types: Type A (medullary thymoma) and Type AB (mixed thymoma) are considered benign. The remainders demonstrate malignant features (1). Cross-sectional imaging helps differentiate thymic tumors from other causes of an anterior mediastinal mass and is essential for accurate staging prior to treatment. Although CT has a limited value in differentiating histological subtypes of thymic tumor, certain findings such as local invasion or pleural seeding are associated with a high recurrence rate (2). 

Accumulation of Tc-99m MIBI in benign and malignant thymomas is previously described (3,4,5). Thymomas are hypercellular lesions. Increased mitochondrial structures in the hypercellular lesions could result in accumulation of MIBI. We would like to remind that during the interpretation of MPS, the raw data should always be reviewed as occasionally valuable additional information on noncardiac pathology may be recognized by extracardiac uptake, as in this case. 

## Figures and Tables

**Figure 1a:**
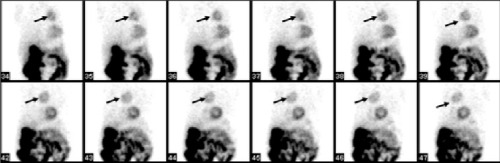
Tc-99m MIBI raw data images. An abnormal focus ofuptake at the upper side of the heart (arrows). Physiological traceruptake is also seen within the heart, stomach, bowel and gallbladder

**Figure 1b:**
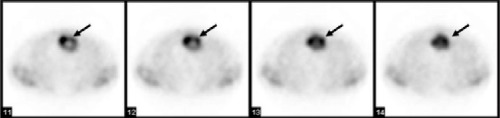
Tc-99m MIBI transaxial images show abnormal increasedactivity located in the anterior mediastinum corresponding to themass revealed on CT in Figure 2
